# Host miRNA-21 promotes liver dysfunction by targeting small intestinal *Lactobacillus* in mice

**DOI:** 10.1080/19490976.2020.1840766

**Published:** 2020-12-10

**Authors:** André A. Santos, Marta B. Afonso, Ricardo S. Ramiro, David Pires, Madalena Pimentel, Rui E. Castro, Cecília M.P. Rodrigues

**Affiliations:** a Research Institute for Medicines (iMed.ULisboa), Faculty of Pharmacy, Universidade de Lisboa, Lisbon, Portugal; b Instituto Gulbenkian de Ciência, Oeiras, Portugal

**Keywords:** Cholestasis, D-lactate, gut microbiota, miRNAs, small intestinal homeostasis

## Abstract

New evidence shows that host-microbiota crosstalk can be modulated via endogenous miRNAs. We have previously reported that miR-21 ablation protects against liver injury in cholestasis. In this study, we investigated the role of miR-21 in modulating the gut microbiota during cholestasis and its effects in liver dysfunction. Mice lacking miR-21 had reduced liver damage and were protected against small intestinal injury as well as from gut microbiota dysbiosis when subjected to bile duct ligation surgery. The unique microbiota profile of miR-21KO mice was characterized by an increase in *Lactobacillus*, a key microbiome genus for gut homeostasis. Interestingly, *in vitro* incubation of synthetic miR-21 directly reduced *Lactobacillus* load. Moreover, supplementation with *Lactobacillus reuteri* revealed reduced liver fibrosis in acute bile duct-ligated mice, mimicking the protective effects in miR-21 knockout mice. D-lactate, a main product of *Lactobacillus*, regulates gut homeostasis that may link with reduced liver fibrosis. Altogether, our results demonstrate that miR-21 promotes liver dysfunction through direct modulation of the gut microbiota and highlight the potential therapeutic effects of *Lactobacillus* supplementation in gut and liver homeostasis.

## Introduction

The communication between liver and gut is vital for overall human homeostasis, with the gut microbiota dysregulation correlating with liver disease.^1,2^ Liver diseases, including alcoholic and nonalcoholic fatty liver diseases, have been shown to associate with small intestine bacterial overgrowth,^3^ increased lipopolysaccharide accumulation and intestinal barrier dysfunction.^4^ On the other hand, the gut microbiota plays a crucial role in bile acid homeostasis, with dysbiosis altering host bile acid metabolism and strongly contributing to liver disease.^5^ Nevertheless, it is still unclear whether dysbiosis actively contributes to or is rather a consequence of liver disease. Similarly, the detailed mechanisms that link dysbiosis to liver damage and vice-versa remain poorly explored.^6^

MicroRNAs (miRNAs) are well known to participate in a variety of biological processes such as cellular differentiation, metabolism, proliferation, immune response, and apoptosis. New evidence has recently shown that extracellular miRNAs exported into the intestinal lumen can directly modulate the gut microbiota composition.^7,8^ In addition, the gut microbiota modulates human circulating miRNAs.^9^ We and others have shown that miRNAs strongly impact liver disease, and miRNA targeting embodies putative therapeutic strategies.^10–14^ In particular, we showed that miR-21 knockout (miR-21KO) mice are protected from liver injury, fibrosis and acute oxidative stress when subjected to common bile duct ligation (BDL)-induced liver injury.^14^ Further, using two mouse models of nonalcoholic steatohepatitis (NASH), lack of miR-21 reduces liver steatosis, inflammation, and fibrosis.^13^ Notably, it was recently demonstrated that miR-21 ablation in mice alters the gut microbiota toward increases in Bifidobacterium and Odoribacter, usually present in the healthy gut, and protects against experimental inflammatory bowel disease.^15^

The small intestine harbors the lowest number of bacteria in the gut. With a harsh environment, characterized by high levels of oxygen, bile acids, and anti-microbial peptides,^16^ the small intestine is essential in bile acid signaling and liver homeostasis.^17^ Moreover, it hosts bacteria known to regulate gut homeostasis, such as *Bifidobacterium* and *Lactobacillus spp*.^18^ Indeed, *Lactobacillus spp*. are widely used as probiotics with beneficial effects in intestinal dysregulation^19^ and liver injury.^20^ *Lactobacillus rhamnosus* GG protects high-fat diet-fed mice against liver fat accumulation,^21^ while *Lactobacillus plantarum* NDC 75017 ameliorates lipopolysaccharide-induced oxidative stress and inflammatory injury in the liver.^22^ This suggests that modulation of the gut microbiota through *Lactobacillus spp*. maybe a viable strategy to treat liver disease.^20,21,23,24^

Here, we sought to understand the role of miR-21 as a gut microbiota modulator and its impact on host homeostasis in the absence of bile acid flow. Specifically, we analyzed small intestinal microbiota of miR-21KO mice subjected to BDL and explored miRNA-driven selection of specific bacteria. Our results show that, in comparison to WT mice, miR-21KO mice had attenuated acute BDL-induced liver damage, partially explained by absence of small intestinal dysbiosis and maintenance of gut homeostasis. Further, we demonstrate that increased levels of small intestinal *Lactobacillus ssp*. tune down BDL-induced liver damage, in part, via D-Lactate production and attenuation of macrophage fibrotic response.

## Results

### miR-21 ablation attenuates liver damage, prevents small intestine permeabilization and maintains gut homeostasis

**Figure 1.**
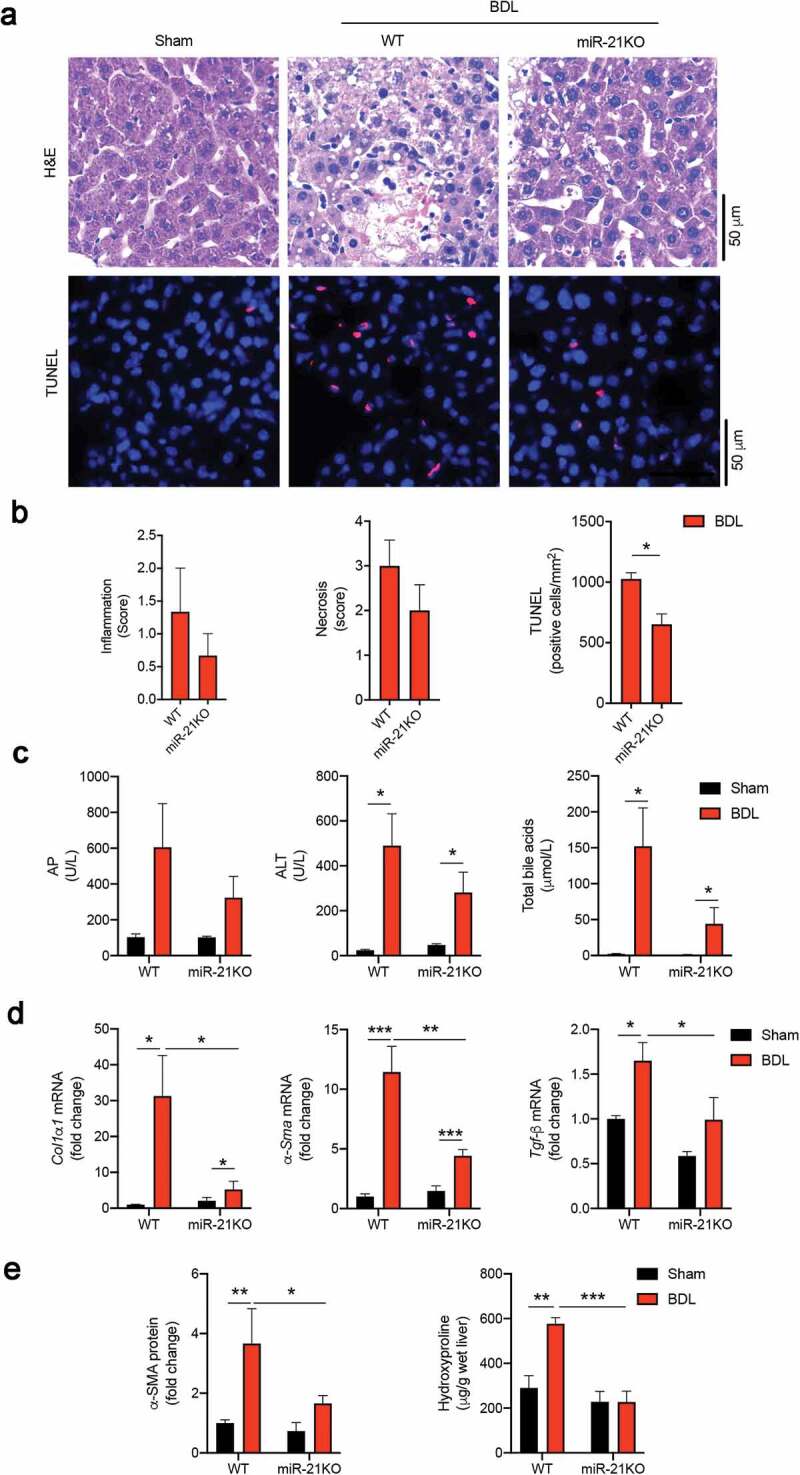
miR-21KO mice are protected from bile duct ligation (BDL)-induced liver injury. (a) Representative images of hematoxylin and eosin (H&E) (*upper panel*) and TUNEL (*lower panel*) stained liver sections after sham operation and in WT and miR-21KO mice 3 days after BDL. Apoptotic cells are shown in red and nuclei are counterstained in blue with Hoechst 33258 dye. Scale bar, 50 μm. (b) Histology scores of inflammation and necrosis, and quantification of TUNEL-positive cells/mm^2^ in WT and miR-21KO mice 3 days after BDL. (c) Serum alkaline phosphatase (AP), alanine aminotransferase (ALT) and total bile acids in WT and miR-21KO mice after either sham operation or BDL for 3 days. (d) liver mRNA expression of *Col1α1, α-Sma* and *Tgf-β* in WT and miR-21KO mice after either sham operation or BDL for 3 days. (e) liver hydroxyproline levels and α-SMA protein in WT and miR-21KO mice after either sham operation or BDL for 3 days. Results are expressed in fold change as mean values with error bars ± SEM of 4–6 individual mice. Data were statistically analyzed with ANOVA Tukey’s multiple comparisons test *p < .05; **p < .01 and ***p < .001

We recently showed that miR-21 is overexpressed in the liver of mice subjected to BDL and that miR-21KO mice display improved bile acid homeostasis and reduced liver damage.^14^ In this study, WT and miR-21KO mice were subjected to sham surgery or BDL for 3 days, after which liver, small intestine tissue, and luminal content were analyzed. Liver histology images and scores as well as TUNEL staining showed overall less severe damage in miR-21KO mice subjected to BDL when compared to WT animals (Figure 1(a–b)). Similarly, mRNA expression levels of *α-Sma* (*p* = .004), *Col1α1* (*p* = .034) and *Tgf-β* (*p* = .047) were significantly decreased in the liver of miR-21KO animals. Corroborating mRNA expression data, hydroxyproline, and α-SMA protein levels confirmed an increase in liver fibrosis after BDL in WT mice (*p* = .0036 and *p* = .0038, respectively), but not in miR-21KO (Figure 1(c–e)). Differences in inflammation between WT and miR-21KO mice after BDL were, however, less evident in agreement with that previously reported.^14^

**Figure 2.**
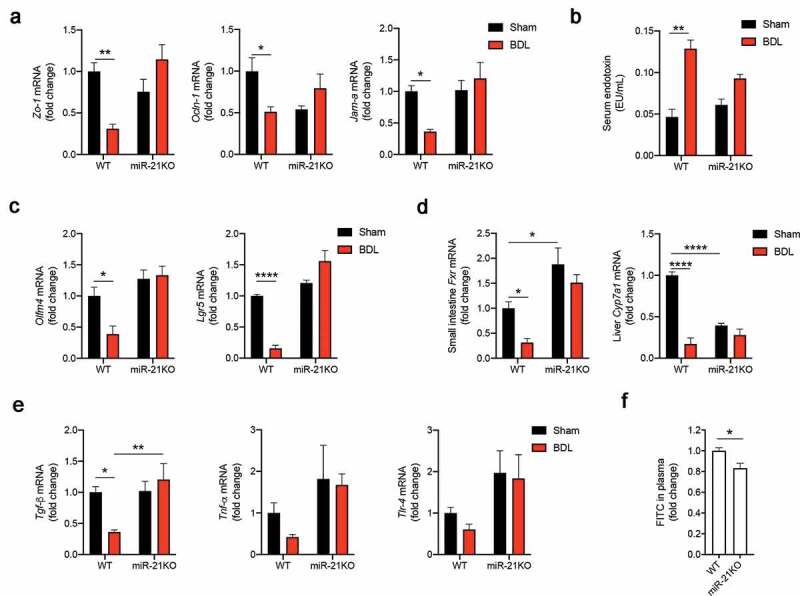
miR-21KO mice are protected from small intestine permeabilization. (a) mRNA expression levels of small intestine tight junctions *Zo-1, Ocldn-1* and *Jam-a* in WT and miR-21KO mice after either sham operation or BDL for 3 days. (b) Serum endotoxin levels in WT and miR-21KO mice after either sham operation or BDL for 3 days. (c) mRNA expression levels of small intestine stem cell markers *Olfm4* and *Lgr5* in WT and miR-21KO mice after either sham operation or BDL for 3 days. (d) mRNA expression levels of small intestinal *Fxr* and liver *Cyp7a1* in WT and miR-21KO mice after either sham operation or BDL for 3 days. (e) mRNA expression levels of small intestinal inflammatory markers *Tgf-β, Tnf-α* and *Tlr-4* in WT and miR-21KO after either sham operation or BDL for 3 days. (f) Analysis of FITC-dextran 4 kDa present in plasma of WT and miR-21KO mice. Results are expressed in fold change as mean values with error bars ± SEM of 4–6 individual mice. Data were statistically analyzed with ANOVA Tukey’s multiple comparisons test with the exception of *Fxr* mRNA and FITC in plasma, which were analyzed by unpaired t-test. *p < .05; **p < .01

Gut permeabilization strongly associates with the gut microbiota dysbiosis and liver disease,^25^ with factors such as tight junctions, stem cell proliferation and gut regeneration being key for maintaining gut wall function. We next determined the effect of miR-21 on the maintenance of intestinal paracellular integrity by measuring mRNA expression of tight-junction proteins *Zo-1*, occludin-1 (*Ocln-1*) and junctional adhesion molecule-A (*Jam-a*). BDL reduced expression of all these genes in WT animals, when compared to mice subjected to sham surgery (*Zo-1, p* = .005; *Ocln-1, p = *.026; and *Jam-a, p* = .04). Conversely, miR-21KO mice kept high levels of these genes, independently of BDL surgery. Interestingly, serum endotoxin levels were almost threefold increased after BDL in WT mice (*p* = .0012) but only 1.5-fold increase (*p* = .0872) in miR-21KO (Figure 2(a)), suggesting a potential direct link between gut dysregulation and liver disease. Similar results were obtained when analyzing the expression of olfactomedin 4 (*Olfm4, p* = .037) and leucine-rich repeat-containing G-protein coupled receptor 5 (*Lgr5, p* < .0001) as measures of intestine stem cell marker status and potential gut regeneration (Figure 2(b)). Moreover, intestinal *Fxr*, a well-known modulator of bile acid synthesis in the liver through cytochrome P450 7A1 (*Cyp7a1*), has been shown to protect against ileum injury induced by BDL.^26^ Of note, mRNA levels of small intestinal *Fxr* and liver *Cyp7a1* were significantly decreased after BDL in WT mice (*p* = .0022 and *p* < .0001, respectively), but maintained constant in miR-21KO animals. In addition, miR-21KO mice exhibited higher levels of *Fxr* and lower expression of *Cyp7a1*, comparing with WT mice (*p* = .0304 and *p* < .0001, respectively) (Figure 2(c)). Finally, increased miR-21 expression has been associated with inflammatory bowel disease,^27^ while *Tgf-β* is thought to modulate gut mucosal regeneration after injury and immune regulation, thus preserving barrier function.^28^ In our model, miR-21KO mice were protected from BDL-induced decrease of *Tgf-β* mRNA levels in small intestine (*p* = .040) (Figure 2(d)). To corroborate these results, we administrated fluorescein isothiocyanate (FITC)-dextran 4-kDa to both WT and miR-21KO mice by oral gavage and collected serum to evaluate FITC in circulation. miR-21KO mice exhibited significantly diminished intestinal permeability when compared to WT mice (*p = *.042) (Figure 2(e)). These results suggest that miR-21KO mice have a less permeable gut, even in the absence of any disease stimuli. Altogether, these results show that miR-21 ablation protected from both liver injury and loss of gut homeostasis.

### miR-21 ablation prevents small intestinal dysbiosis and enables Lactobacillus growth

**Figure 3.**
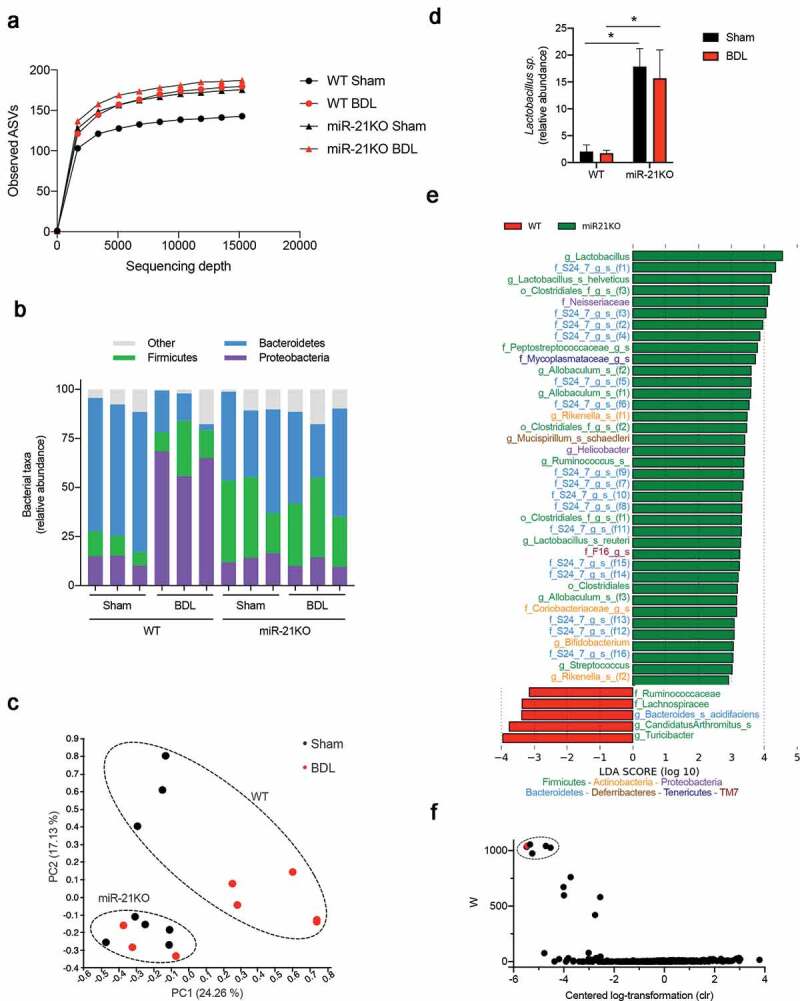
miR-21KO mice are protected from small intestinal dysbiosis. (a) Alpha diversity measured by observed OTU in WT and miR-21KO mice after either sham operation or BDL for 3 days. (b) Relative abundance of bacterial phyla in WT and miR-21KO mice after either sham operation or BDL for 3 days. (c) Principal component analysis (PCoA) of the β-diversity metric Bray-Curtis with PC1 and PC2 separating WT (black, Sham; red, BDL) and miR-21KO (black, Sham; red, BDL) mice after either sham operation or BDL for 3 days. (d) Relative abundances of *Lactobacillus spp*. in WT and miR-21KO mice after either sham operation or BDL for 3 days, mean values were calculated as fold change versus sham WT with error bars ± SEM of 4–6 individual mice and statistical analysis performed with ANOVA Tukey’s multiple comparisons test. *p < .05. (e) Linear discriminant analysis effective size (LEfSe) with Kruskal-Wallis test among classes and Wilcoxon test between subclasses. Taxa shown were significantly different between WT and miR-21KO mice after either sham or BDL for 3 days (*p* < .05). (f) Analysis of composition of microbiomes (ANCOM) between WT and miR-21KO mice after either sham or BDL 3 days (dash circle identifies the five significantly different features across the X and Y groups; red-marker indicates *Lactobacillus helveticus* feature)

Mice subjected to BDL surgery develop the gut microbiota dysbiosis associated with disruption of the bile acid pool.^29^ Since miR-21 is present in mice and human small intestinal lumen,^7^ we tested the hypothesis that miR-21 ablation impacts BDL-induced the gut microbiota dysbiosis. The gut microbiota composition of miR-21KO and WT mice were evaluated by sequencing the 16S rRNA gene from small intestine lumen samples and analysis using QIIME2 software. Although not statistically different, alpha diversity analysis showed increased number of amplicon sequence variants (ASVs) in WT mice after BDL. Conversely, this parameter was not affected in miR-21KO mice, as these animals already have increased ASVs prior to BDL (Figure 3(a)). At the taxonomic level, absence of miR-21 in sham mice shifted the relative abundance of dominant bacterial phyla. The gut microbiota of sham WT mice was strongly enriched in Bacteroidetes (~70%), while a balanced proportion of Firmicutes (~38%) and Bacteroidetes (~36%) was observed in sham miR-21KO mice (Figure 3(b)). Further, BDL dramatically impacted small intestine microbiota composition in WT mice, promoting a ~ fourfold increase in Proteobacteria (*p* < .0001) and a ~ fivefold decrease in Bacteroidetes abundance (*p* < .0001). Interestingly, the specific gut microbiota of miR-21KO mice was not disturbed by BDL, suggesting that miR-21 deletion both shapes and stabilizes microbiota abundance. In agreement, principal coordinate analysis using Bray-Curtis as the β-diversity metric showed that the first two principal components explained >40% of the variation, clearly separating WT and miR-21KO mice. Moreover, while WT mice subjected to BDL or sham clustered separately, the same was not true for miR21-KO mice (Figure 3(c)). Strikingly, the relative abundance data, showed that *Lactobacillus spp*. was significatively different between WT and miR-21KO mice, with a ~ sixfold increase in miR-21KO compared with WT animals, irrespective of surgery (sham WT versus miR-21KO mice, *p* = .021; and BDL WT versus miR-21KO mice, *p* = .030) (Figure 3(d)). To evaluate all taxa that were driving separation between WT and miR-21KO samples, whether sham or BDL, we performed a linear discriminant analysis effect size (LEfSe). This validated the genera *Lactobacillus* as the taxa that most strongly discriminated between miR-21KO and WT mice (Figure 3(e)). To corroborate the LEfSe results, we performed an analysis of composition of microbiomes (ANCOM) between WT and miR-21KO mice. Five features presented a W > 1000, being overrepresented in miR-21KO mice (figure 3(f)). Four features belonged to the S24-7 family (confidence of 99%) and one feature was specific to the *Lactobacillus helveticus* species (confidence of 73%) (Supplementary Table S1). Finally, cohousing experiments confirm that the gut microbiota composition in miR-21KO mice was not due to cage effects nor to independent breeding for a few generations. WT and miR-21KO animals were cohoused for 1 month and then individualized into different cages for one additional month (Fig. S1A). The results showed that cohoused miR-21KO mice displayed similar relative abundance of *Lactobacillus spp*. comparing with cohoused WT mice. Remarkably, after single housing, miR-21KO animals recovered the higher amounts of *Lactobacillus spp*. when compared to single or cohoused WT mice (*p* = .0119 and *p* = .0204, respectively) (Fig. S1B). These results show that the absence of miR-21 increases *Lactobacillus* relative abundance in the small intestine, suggesting a contribution toward reduced liver damage.

### Lactobacillus reuteri is susceptible to synthetic miR-21

**Figure 4.**
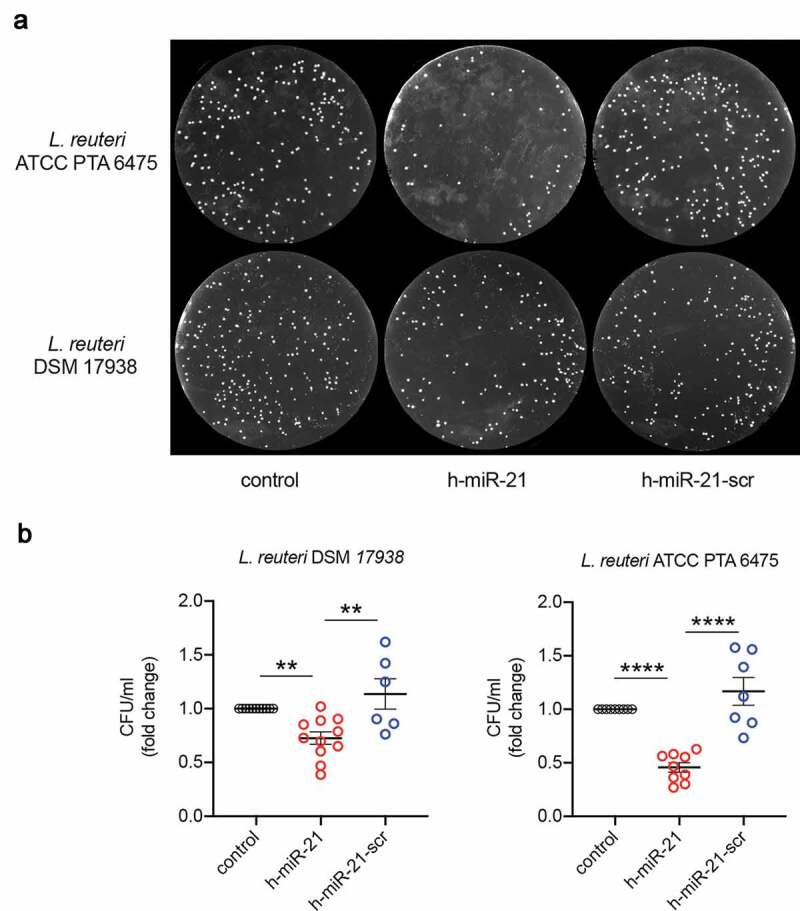
*Lactobacillus spp*. are susceptible to synthetic miR-21. (a) Bacterial colonies of *L. reuteri* DSM 17938 and *L. reuteri* ATCC PTA 6475 exposed to either sterile water (control), synthetic miR-21 (h-miR-21) or synthetic scrambled miR-21 (h-miR-21-scr). (b) Colony-forming unit (CFU)/ml of *L. reuteri* DSM 17938 and *L. reuteri* ATCC PTA 6475 exposed to either sterile water (control), synthetic miR-21 (h-miR-21) or synthetic scrambled miR-21 (h-miR-21-scr). Representative numbers of bacterial colonies cultured on MRS agar. Mean values were calculated as fold change versus controls with error bars ± SEM of 6–10 individual experiments. Statistical analysis performed with ANOVA Tukey’s multiple comparisons test. **p < .01, ****p < .0001

To investigate the direct impact of miR-21 on *Lactobacillus* growth, we developed an *in vitro* assay using two strains of *Lactobacillus: L. reuteri* DSM 17938 and *L. reuteri* ATCC PTA 6475. Both strains were incubated with either a synthetic human miR-21 sequence (h-miR-21) or a scramble miR-21 sequence (h-miR21-scr). h-miR21 significantly diminished the number of colony-forming units in both *L. reuteri* strains when compared to the control plates (*p < *.01) (Figure 4(a,b)). This effect is specific, as h-miR21-scr failed to inhibit the growth of both strains. Thus, miR-21 present in the small intestinal lumen may contribute to modulate *Lactobacillus spp*. in WT mice.

### Administration of Lactobacillus reuteri attenuates BDL-induced liver damage in mice

**Figure 5.**
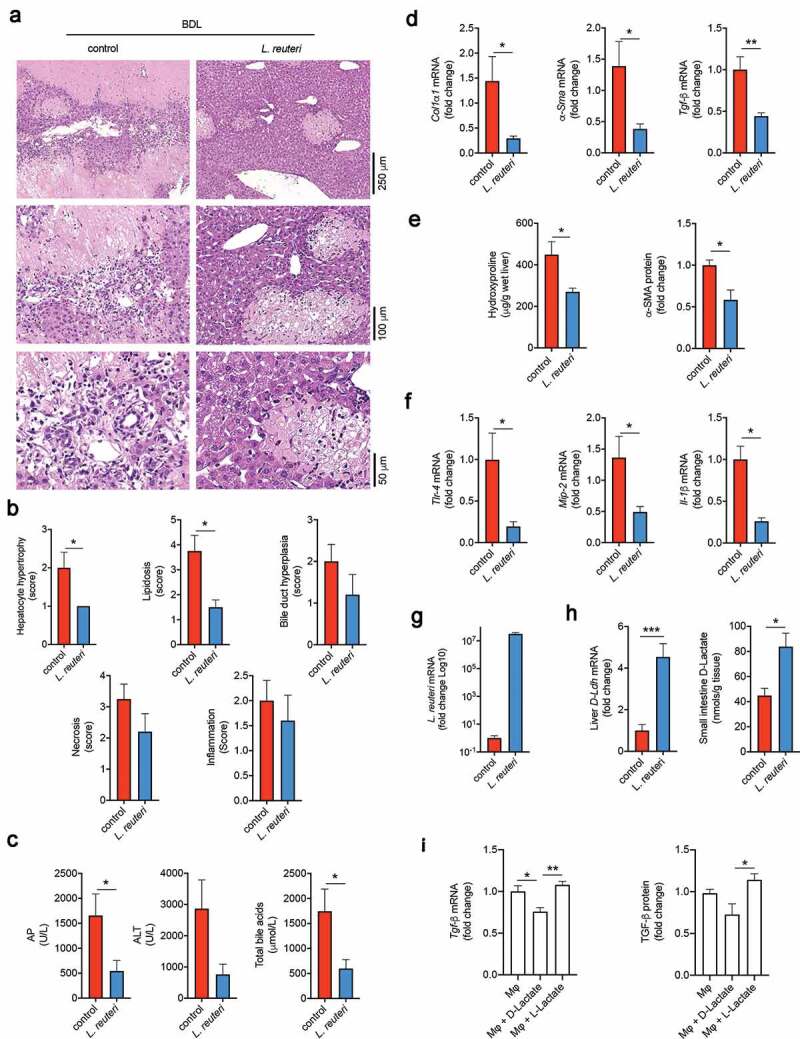
*Lactobacillus reuteri* supplementation protects from bile duct ligation (BDL)-induced liver damage. (a) Representative images of hematoxylin and eosin (H&E) stained liver sections from control and *Lactobacillus reuteri* DSM 17938 (*L. reuteri*) supplemented mice after BDL for 3 days. (b) Histology scores for hepatocyte hypertrophy, lipidosis, bile duct hyperplasia, necrosis and inflammation in control and *L. reuteri* supplemented mice 3 days after BDL. (c) Serum alkaline phosphatase (AP), alanine aminotransferase (ALT) and total bile acids in control and *L. reuteri* supplemented mice 3 days after BDL. (d) mRNA expression of liver fibrosis markers *Col1α1, α-Sma* and *Tgf-β* in control and *L. reuteri* supplemented mice after BDL for 3 days. (e) Liver hydroxyproline and α-SMA protein levels in control and *L. reuteri* supplemented mice after BDL for 3 days. (f) mRNA expression levels of liver inflammatory markers *Tnf-α, Il-1β, Mip-2* and *Tlr-4* in control and *L. reuteri* supplemented mice after BDL for 3 days. (g) qPCR mRNA expression levels of *L. reuteri* in small intestinal lumen samples of control and *L. reuteri* supplemented mice after BDL for 3 days. (h) qPCR mRNA expression levels of liver D-Lactate dehydrogenase (*D-Ldh*) in control and *L. reuteri* supplemented mice after BDL for 3 days. (i) mRNA expression and protein levels of TGF-β in mouse macrophages stimulated with 1 mM of either D-Lactate or L-Lactate. Mean values of the *in vivo* experiments were calculated as fold change versus control with error bars ± SEM of 4–6 individual mice. Statistical analysis performed using unpaired t-test. Mean values of the *in vitro* assays were calculated as fold change versus control macrophages with error bars ± SEM of 5 individual experiments. Statistical analysis was performed with ANOVA Tukey’s multiple comparisons test. *p < .05, **p < .01 and ***p < .001

To evaluate the role of increased gut *Lactobacillus* in liver disease, mice had free access to water supplemented with *L. reuteri* DSM 17938 one week prior to BDL surgery and for the next 3 days after BDL. Overall, in control mice, necrosis was marked, multifocal to coalescent, with moderate bile duct hyperplasia and inflammatory cell infiltration. On the other hand, multifocal necrosis and inflammatory cell infiltration were mild after *L. reuteri* administration (Figure 5(a)). In fact, *L. reuteri* supplementation significantly reduced scores of hepatic hypertrophy (*p* = .049) and lipid accumulation (*p* = .018), liver enzyme AP (*p* = .042), ALT (*p* = .050) and total bile acids (*p* = .035) as well as mRNA levels *α-Sma* (*p* = .026), *Col1α1* (*p* = .034) and *Tgf-β* (*p* = .006). Liver hydroxyproline (*p* = .032) and α-SMA protein analysis (*p* = .035) corroborated the mRNA data and confirmed that *L. reuteri* supplementation attenuates acute BDL-induced liver fibrosis (Figure 5(b–e)). Further, inflammatory mRNA markers were decreased, including *Mip-2* (*p* = .028), *Tlr-4* (*p* = .027) and *Il-1β* (*p* = .002) (figure 5(f)). Finally, q-PCR analysis of *L. reuteri* 16S RNA confirmed the presence of *L. reuteri* in supplemented mice (Figure 5(g)). These results showed that *Lactobacillus* treatment improved the hepatic outcome of BDL, while corroborating the beneficial effect of increased *Lactobacillus* load in miR-21KO mice.

Indeed, increasing amounts of *Lactobacillus* in the gut microbiota may contribute to attenuate liver disease via increased short-chain fatty acids (SCFA)^30^ and, in particular, via production of D-Lactate by bacteria,^31^ which is hydrolyzed in mammal cells by the mitochondrial D-lactate dehydrogenase (*D-Ldh*).^32^ This SCFA is an important energy substrate for liver mitochondria^33^ and may inhibit macrophage pro-inflammatory response.^34^ Although D-lactate was not detectable in serum or liver tissue, supplementation with *L. reuteri* induced a fourfold increase of hepatic *D-Ldh* mRNA. Further, increased D-lactate levels in the small intestine tissue (*p = *.0181) confirmed increased production in the gut (Figure 5(h)). Interestingly, *in vitro* experiments showed that D-lactate but not L-lactate can significantly reduce macrophage *Tgf-β* mRNA expression (*p* = .004) and protein production (*p* = .0124) in the absence of any inflammatory stimulus (Figure 5(i)). Thus, these results suggest that supplementation with *L. reuteri* and subsequent D-lactate production may protect against BDL-induced liver damaged.

## Discussion

**Figure 6.**
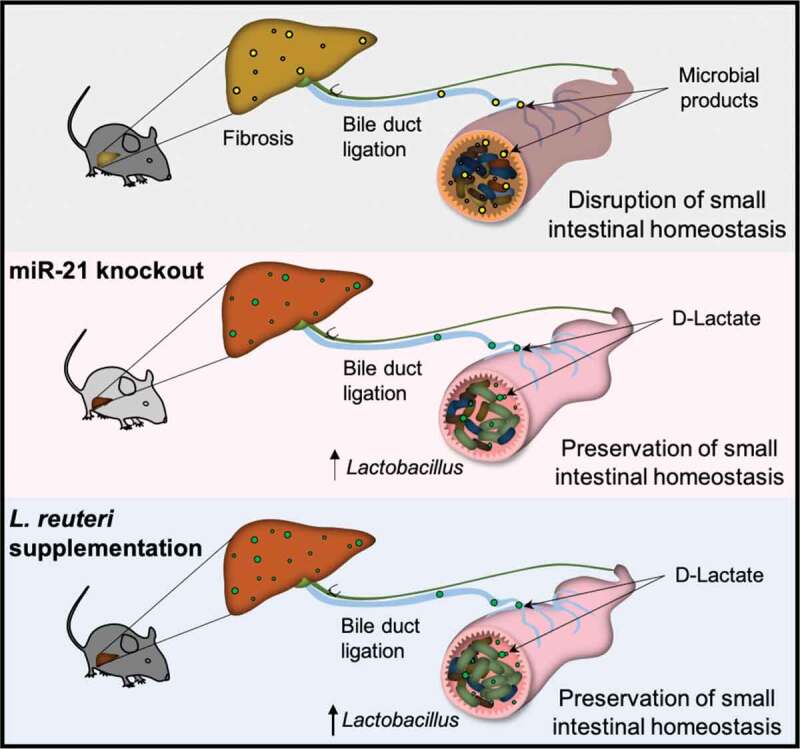
Schematics showing that miR-21KO mice are protected from acute BDL-induced liver damage partially by absence of small intestinal dysbiosis and maintenance of gut homeostasis. Further, increased levels of small intestinal *Lactobacillus ssp*. tune down BDL-induced liver damaged in part via D-Lactate production and attenuation of macrophage fibrotic response

Dysregulation of the gut and liver crosstalk strongly impacts liver disease.^35^ For instance, bile acids are produced in the liver as primary bile acids and metabolized in the gut to secondary bile acids. Any alteration in either organ may impact on the bile acid pool and thus influence the host overall homeostasis.^36–38^ miRNAs play a crucial role in regulating gene expression and may potentially modulate the gut microbiota.^7^ A recent study showed that fecal transplantation from miR-21KO to germ-free mice protects from inflammatory bowel disease.^15^ Our current study shows that genetic ablation of miRNA-21 improves gut and liver homeostasis and modulates small intestine microbiota composition toward increased *Lactobacillus* load. Further, oral supplementation with *Lactobacillus* confers protection against acute cholestasis (Figure 6).

We have previously shown that miR-21 ablation protects from increased liver injury induced by BDL.^14^ This is an interesting model to evaluate liver and gut crosstalk. On one hand, accumulation of bile acids in the liver will promote cellular damage.^39^ On the other hand, the blockage of bile acid flow will alter the small intestine and promote gut microbiota alterations that may further impact on liver damage. Here, we report that miR-21 dysregulates small intestinal homeostasis and directly inhibits *Lactobacillus spp*. growth. Interestingly, while alpha diversity was not significantly different between treatment groups, the number of ASVs was higher in miR-21KO prior to BDL and the surgical procedure increased the alpha diversity of WT mice to a level similar to that of miR-21KO animals. Moreover, we show that the gut microbiota from miR-21KO sham mice display an increased Firmicutes/Bacteroidetes ratio mostly attributed to augmented *Lactobacillus spp*. relative abundance, and this profile is not affected by BDL. The effect of systemic miR-21 absence, resulting in increased *Lactobacillus spp*., was confirmed in cohousing experiments. After cohousing miR-21KO and WT mice for 1 month, no differences were found in the relative abundance of *Lactobacillus* between the two genetic backgrounds. This may result from passage of miR-21 and other host/microbiota modulators from WT to miR-21KO animals – and vice-versa – through coprophagy. However, when mice were isolated in individual cages for an additional month, miR-21KO mice presented again with increased *Lactobacillus* relative abundance. This suggests that the absence of miR-21 quickly drives microbiota to increased abundance of *Lactobacillus*. We should emphasize that the cohousing experiment aimed only to access the ability of the genetic background of miR-21KO mice to generate a conducive environment for *Lactobacillus* spp. growth, thus not accounting for other cage-based effects caused by a number of other factors. The direct modulation of *Lactobacillus* by miR-21 was elucidated in *in vitro* assays. When synthetic miR-21 but not scrambled miR-21 reduced the number of CFUs when added to two different *L. reuteri* strains. These data confirms that miR-21 can target gut *Lactobacillus*, impairing bacterial growth, thus strongly suggesting that genetic ablation of miR-21 in mice favors the relative abundance of *Lactobacillus* in the small intestinal lumen. Still, miR-21 could also indirectly shape the gut microbiota in a host cell-autonomous manner. Nevertheless, since miR-21KO mice cohoused with WT mice only showed a tendency to increased *Lactobacillus spp*. abundance, the impact in the modulation of microbiota by the intricate effect of miR-21 deletion in host cells is likely less relevant than that of the direct effect of miR-21 in *Lactobacillus*. Modulation of the gut microbiota by miRNAs remains a poorly explored concept. Nonetheless, recent data suggests that intestinal lumen miR-515-5p and miR-1226-5p favor growth of *Fusobacterium spp*. and *Escherichia coli* in mice.^7^ In addition, BDL resulted in a shift toward increased amounts of Proteobacteria in the gut microbiota of WT mice, previously implicated in the development of liver disease through increased liver fibrosis and inflammation.^29^ Here, we show that in miR-21KO mice were protected from BDL-induced dysbiosis and associated with a unique microbiota profile, underscoring the significance of miR-21 in the modulation of the small intestine gut microbiota.

In parallel with bile acid pool dysregulation, BDL-induced injury further associates with gut permeabilization.^29^ In this regard, we showed that miR-21 ablation reduces intestinal permeability coupled with increased small intestine *Tgf-β* mRNA expression. Indeed, it is known that *Lactobacillus spp*. induces small intestinal *Tgf-β*, thought to prone the immune system against bacterial dysregulation^40–42^ and to contribute for the maintenance of barrier function.^28,43^ Additionally, the absence of miR-21 sustained *Lgr5* and *Olfm4* levels after BDL surgery, potentially favoring small intestine homeostasis through stem cell mucosa integrity maintenance. In fact, probiotic strain *L. reuteri* D8 has been shown to protect small intestine barrier through activation of epithelial proliferation via *Lgr5*.^44^ Apart from reduced intestinal permeability, miR-21KO mice also exhibited increased small intestine *Fxr* expression. These results are in agreement with previous studies showing that increased *intestinal Fxr* correlates with protection from BDL-induced fibrosis.^45^ Moreover, *Cyp7a1* is regulated inversely *to Fxr* in miR-21KO mice, which is in agreement with a tight regulation of bile acid synthesis.^46^ It was recently shown that *Mdr2* knockout mice, that spontaneously develop liver primary sclerosing cholangitis, also exhibit decreased liver *Cyp7a1* and increased small *intestine Fxr* mRNA when treated with a *Lactobacillus* strain, corroborating a link between *Lactobacillus* and *Fxr* and *Cyp7a1* modulation that further impacts bile acid homeostasis.^47^

Considering our results with miR-21KO mice and the potential role of *Lactobacillus* in the reduction of BDL-induced liver injury and fibrosis, we evaluated the effect of *Lactobacillus reuteri* DSM 17938 supplementation as a preventive therapeutic strategy in experimental acute cholestasis. *Lactobacillus* strains have already been used in randomized-controlled trials as probiotics, and proved to ameliorate liver disease outcomes,^48^ Here, we show that the use of probiotic *L. reuteri* DSM 17938 in BDL mice ameliorates liver disease through decreased fibrosis, underscoring the protective effect of increased *Lactobacillus* load in the liver of miR-21KO mice. We should emphasize that altering the gut microbiota has a panoply of effects in gut pathophysiology, including changes in permeabilization, bacterial communities and SCFAs, all influencing gut-liver overall homeostasis.^49^ Our results demonstrate that increasing small intestinal *Lactobacillus* contributes to attenuate liver disease. In parallel with general immunologic effects in the gut and modulation of bile acid synthesis via *Fxr, Lactobacillus* contributes toward diminished liver fibrosis, where D-Lactate may embody an important mitochondrial energy source, particularly in the liver,^33^ and modulate macrophage anti-inflammatory response.^50^

In conclusion, we provide evidence that both miR-21 ablation and supplementation with *L. reuteri* contribute to reduced liver injury in mice after BDL through maintenance of gut homeostasis impacting on acute liver fibrosis induced by BDL. Thus, miRNAs may a useful tool to specifically target a bacterium. Further studies should pinpoint the underlying mechanisms of crosstalk between miRNAs, small intestinal *Lactobacillus* and the liver.

## Materials and methods

### Animal experiments

The surgical procedure for common bile duct ligation (BDL) was performed in 8–10-week old wild type C57BL/6NCrl female mice (WT) (Charles River laboratory) with 25–30 g or in miR-21-deficient mice (miR-21KO; C57BL/6NCrl miR-21 *loxP/loxP* mice; UT Southwestern Medical Center) as previously described.^14^ The phenotype remained completely stable over all experiments performed in different experimental groups. Controls underwent sham operation with exposure of the common BDL without ligation. Four to six animals were included in each experimental group. Surgeries were performed as described previously.^51^ Briefly, the procedure started with mice anesthetized with isoflurane, laid on a heating pad followed by disinfection of the abdominal skin. Next, to expose the xyphoid process, a 3 cm long incision was performed in the midline of the abdominal skin. After the laparotomy, the skin was retracted bilaterally to expose the liver. Using microdissection forceps, a 5–0 non-absorbable synthetic monofilament was positioned around the bile duct and closed. Middle and left liver lobes were gently placed into their original location and the abdominal wall muscle closed with synthetic non-absorbable monofilament sutures. Finally, after closing the skin with wound clips, mice recovered for 15 min in a warming pad. To minimize post-operating pain, analgesic buprenorphine (0.05 mg/kg body weight) was subcutaneously administered before surgery and 48 h after surgery. Three days after surgery, animals were euthanized with isoflurane overdose between 10:00 AM and 14:00 PM without a fasting period. Serum was collected for the evaluation of alanine aminotransferase (ALT) and alkaline phosphatase (AP) (ABX ALT and AP assay kit; Horiba) and total bile acids (3α-hydroxysteroid dehydrogenase enzymatic assay kit; Randox Reagents). Serum endotoxin levels were measured using the ToxinSensor Chromogenic LAL Endotoxin Assay Kit (GenScript). Liver total collagen was measured by colorimetric determination of the collagen-specific amino acid hydroxyproline using the hydroxyproline assay kit (Sigma-Aldrich). All kits were used accordingly to manufacturers’ protocols. Total small intestinal lumen content was collected and stored in liquid nitrogen for microbiota analysis. The ileum section of the small intestine was collected, rinsed in normal saline and immediately flash-frozen in liquid nitrogen for RNA extraction. The liver was also removed; one lobe was collected, rinsed in normal saline and immediately flash-frozen in liquid nitrogen for RNA extraction; the other lobe was fixed in paraformaldehyde (4%, wt/vol) in phosphate-buffered saline (PBS; Thermo Fisher Scientific, no. 10010031) for paraffin-embedded sectioning.

For supplementation with *Lactobacillus* BDL experiments, 8–10-week old C57BL/6 female mice with 25–30 g had free access to the supplemented water during one week before BDL and until the euthanasia. *Lactobacillus reuteri* DSM 17938 (BioGaia Probiotics) were grown for 16 h at 37 °C in De Man, Rogosa, and Sharpe (MRS) broth (Sigma-Aldrich) in aerobic conditions. Next, cells were centrifuged at 2000 *g*, washed with sterile water, resuspended in sterile water to 10^8^ cells/mL, and the water solution was changed every 3 days.

Finally, to test the cage coprophagy effect and assign the effect of miR-21 to *Lactobacillus spp*. we performed a cohousing experiment. Four cages containing two WT and two miR-21KO mice were fed normal diet for 1 month. Next, one WT and one miR-21KO were sacrificed and their small intestine luminal samples stored at −80 °C until bacterial DNA extraction. The remaining animals were separated into eight different cages in which each cage contained only one mouse, either WT or miR-21KO. After 1 month, mice were sacrificed, and small intestine luminal samples stored at −80 °C until bacterial DNA extraction (Fig. S1A).

### Liver histology

Liver samples were immersion fixed in 10% neutral-buffered formalin, routinely processed for paraffin embedding, sectioned at 4 µm, and stained with hematoxylin and eosin (H&E). Lesions were examined by an experienced veterinary pathologist blinded to experimental groups and classified according to previously published criteria (INHAND, International Harmonization of Nomenclature and Diagnostic Criteria for Lesions in Rats and Mice). In brief, a semi-quantitative score was determined for several hepatic lesions (hepatocellular damage and necrosis, inflammatory cell infiltration, lipidosis, and bile duct hyperplasia) according to a 5-tier severity scale: 0, absent; 1, minimal; 2, mild; 3, moderate; 4, marked. Representative photographs were acquired using NDP.view2 software in slides digitally scanned in the Hamamatsu NanoZoomerSQ (Hamamatsu).

Terminal deoxynucleotidyl transferase dUTP nick end labeling (TUNEL) assay was performed in 5 μm liver tissue cryosections using the ApopTag® Red *In Situ* Apoptosis Detection Kit, according to manufacturer’s instructions (Merck Millipore). Nuclei were counterstained with Hoechst 33258 (Sigma-Aldrich) at 50 μg/mL in PBS for 10 min at room temperature. Six images per sample were obtained and fluorescent red nuclei were considered TUNEL-positive cells. Data are expressed as the number of TUNEL-positive cells per mm^2^.

### 16S sequencing and analysis

Bacterial DNA was extracted from small intestinal lumen content using a QIAamp Fast DNA Stool Mini Kit (Qiagen), following manufacturer’s instructions. The gut microbiota composition of small intestinal lumen samples was determined by sequencing the V4 region of the 16S rRNA gene (primers 515 F-806 R) using a 280-multiplex approach on a 2x250bp PE MiSeq run (Illumina, Inc.) and analyzed with QIIME2 software.^52–58^ Demultiplexed paired-end reads (fastq files) were denoised with DADA2. Taxonomy assignment of ASVs (amplicon sequence variant) was done against the Greengenes database using Classify-sklearn. ASVs sequences were aligned and phylogenetically uninformative positions were masked, before creating a maximum-likelihood phylogenetic tree with FastTree. For all analysis, we rarefied to the minimum coverage across all samples (i.e. 15238 reads). Rarefaction curves confirmed that alpha diversity had already saturated at this coverage level (Figure S3A). For alpha diversity analysis, the following indexes were used: Shannon index (quantitative community richness), Observed ASVs (qualitative community richness) and Faith’s Phylogenetic Diversity (qualitative community richness with phylogenetic relationships) and Pielou’s evenness (to evaluate equitability). Beta diversity analysis was performed to evaluate dissimilarities in microbial communities between groups using Bray–Curtis distances (quantitative community dissimilarity). Distances matrices were then clustered using principal coordinate analysis (PCoA) and PERMANOVA to test for differences between groups. Moreover, we used betadisper to test for homogeneity of variances as this is an assumption of PERMANOVA. LEfSe analysis was used to identify taxa that were different between groups. This was done through the Galaxy server from Huttenhower lab, with the standard specifications.^59^ For cohousing experiments, we rarefied to the minimum coverage across all samples (i.e. 21276 reads). Rarefaction curves confirmed that alpha diversity had already saturated at this coverage level (Figure S3B).

### FITC-Dextran permeability assay

This procedure was performed using three WT and three miR-21KO mice. Four hours before gavage with Fluorescein isothiocyanate-dextran (FITC-dextran) 4 kDa (Sigma-Aldrich, no. 46944), food was removed. Animals were gavaged with 200 µL of FITC-Dextran (0.8 mg/mL solubilized in PBS). After gavage, mice were left in their cages without water or food for 4 hours. Next, mice were sacrificed, and blood recovered directly from the heart into a 1.5 mL plastic tube already containing 5 µL of heparin. Blood was then centrifuged, and plasma collected. A calibration curve for FITC-Dextran (0, 0.25, 0.5, 0.75, 1, 1.25, 1.5, 1.75, 2, 3, 4 µg/mL) was prepared. Per sample, 12.5 µL of PBS were added to 12.5 µL of plasma. Fluorescent FITC-4kDa Dextran was measured with 485 mm excitation and 535 mm emission using the GloMax-Multi+Detection System (Promega).

### D-Lactate treatment of macrophages

The mouse macrophage cell line J774A.1 (ATCC, no. TIB-67) was grown in Dulbecco’s Modified Eagle’s Medium supplemented with 2 mM HyClone L-glutamine, 1 mM HyClone 100 mM sodium pyruvate solution, 10 mM HyClone HEPES buffer (Thermo Fisher Scientific, no. SH30237.01) and 10% HyClone fetal bovine serum (FBS; Thermo Fisher Scientific, no. SV30160.03), and kept at 37 ºC, 5% CO_2_. One day prior to treatments, cells were seeded in 24-well plates in order to achieve 2 × 10^5^ cells per well on the next day. Macrophages were treated with 1 mM of sodium D-lactate or 1 mM sodium L-lactate (Sigma-Aldrich, no. 71716–1 G and 71718–10 G, respectively) and harvested 24 h later for total RNA isolation.

### Real-time RT-PCR

Total RNA was extracted from ileum, liver samples, and J774A.1 macrophages using Trizol Reagent (Thermo Fisher Scientific, no. 15596018), following the manufacturer’s protocol. Total RNA was converted into cDNA using NZY Reverse Transcriptase (NZYTech, no. MB12402), according to the manufacturer’s instructions. Real-time PCR was performed in the QuantStudio™ 7 Flex Real-Time PCR System (Thermo Fisher Scientific). Primer sequences are listed in Table S2. Two independent reactions for each primer set were performed in a total volume of 12.5 μL containing 2x sensiFAST SYBR Hi-ROX kit (Bioline, no. BIO-92020) and 0.6 μM of each primer (Stabvida). The relative amounts of each gene were calculated based on standard curves normalized to the level of HPRT and expressed as fold change from sham WT controls. To quantitate the relative amounts of miR-21, total RNA was converted into cDNA using the TaqMan MicroRNA Reverse Transcription kit (Thermo Fisher Scientific, no. 4366597), according to the manufacturer’s instructions. TaqMan Universal Master Mix II, no UNG and TaqMan MicroRNA assay miR-21 and U6 (Thermo Fisher Scientific) were used for the real-time PCR. The relative miRNA expression levels were normalized to U6 expression. Relative amounts of miR-21 and each gene were determined by the threshold cycle (2^−ΔΔCt^) method.

### Lactobacillus reuteri growth

Two strains of Lactobacillus obtained from BioGaia Probiotics were used: *L. reuteri* DSM 17938 and *L. reuteri* ATCC PTA 6475. Both strains were grown aerobically ON in MRS broth for 16 h at 37 °C, and a 4 × 10^6^ dilution was further used. miRNAs were synthetized either with the human miR-21 sequence (h-miR21), hsa-miR-21-5p sequence 5ʹ uag cuu auc aga cug aug uug a 3ʹ, obtained from www.mirbase.org; or a scramble miR-21 sequence (h-miR21-scr), scrambled sequence 5ʹ cau aua uuu gga gga ugu agc c 3ʹ (Stabvida). Synthetic miRNAs were diluted to the desired concentration in nuclease-free water. 5 µg of synthetic miR were added to each 50 µL of diluted bacterial solution and incubated aerobically for 2.5 h at 37 °C. The mixtures were spread on MRS agar plates in anaerobic jars for 16 h at 37 °C. Total colony-forming units (CFUs) were counted and the results expressed as CFU/mL per plate.

### Data analysis

Statistical analysis was performed with GraphPad Prism 8 software using the following tests: student’s t-test or Mann-Whitney and one-way analysis of variance (ANOVA) with correction of multiple comparisons analysis using statistical hypothesis Tukey when appropriated. Values of *p* < .05 were considered statistically significant. Error bars indicate mean ± standard error of the mean (SEM).

## Supplementary Material

Supplemental Material

## Abbreviations

α-SMA: alpha-smooth muscle actin
ALT: alanine aminotransferase
AP: Alkaline phosphatase
BDL: bile duct ligation
Col1α1: Collagen1α1
CYP7A1: cytochrome P450 family 7 subfamily A member 1
D-LDH: D-lactate dehydrogenase
FITC-Dextran: fluorescein isothiocyanate-dextran 4 kDa; FXR, farnesoid X receptor
HPRT: hypoxanthine phosphoribosyltransferase
Il-1β: interleukin-1β
JAM-A: junctional adhesion molecule A
LGR-5: leucine-rich-repeat-containing G-protein-coupled receptor 5
*L. reuteri*: *Lactobacillus reuteri* DSM 17938; MØ, macrophage
MIP-2: macrophage inflammatory protein 2; miRNA-21, microRNA-21-5p
miR-21KO: miRNA-21 knockout
Ocln-1: Occludin-1
Olfm-4: olfactomedin-4
PCoA: principal coordinates analysis
TGF-β: tumor growth factor-β
TLR-4: toll like receptor-4
TNF-α: tumor necrosis factor-α
WT: wild type
ZO-1: zonula ocludens-1
