# Supplementary material for: Host miRNA-21 promotes liver dysfunction by targeting small intestinal Lactobacillus in mice
Source: Gut Microbes. 2020 Dec 10;12(1):1840766. doi: 10.1080/19490976.2020.1840766 (PMC7733982; doi:10.1080/19490976.2020.1840766)
Supplement: Supplemental Material [file KGMI_A_1840766_SM7709.docx]

**Supplementary Information**

**Host miRNA-21 promotes liver dysfunction by targeting small intestinal *Lactobacillus* in mice**

André A. Santos^a*^, Marta B. Afonso^a^, Ricardo S. Ramiro ^b^,^2^ David Pires ^a^, Madalena Pimentel^a^, Rui E. Castro^a^, and Cecília M.P. Rodrigues^a*^

*^a^Research Institute for Medicines (iMed.ULisboa), Faculty of Pharmacy, Universidade de Lisboa, Lisbon, Portugal; ^b^Instituto Gulbenkian de Ciência, Oeiras, Portugal*

*corresponding authors:

André A. Santos, PhD. Faculty of Pharmacy, Universidade de Lisboa, Av. Prof. Gama Pinto, 1649-003 Lisbon, Portugal; E-mail: [afasantos@ff.ulisboa.pt](mailto:afasantos@ff.ulisboa.pt); Tel: +351 21 7946400

Cecília M. P. Rodrigues, PhD. Faculty of Pharmacy, Universidade de Lisboa, Av. Prof. Gama Pinto, 1649-003 Lisbon, Portugal; E-mail: [cmprodrigues@ff.ulisboa.pt](mailto:cmprodrigues@ff.ulisboa.pt)

**Supplementary Tables**

**Table S1.** Analysis of composition of microbiomes. Features present in the Analysis of Composition of Microbiomes (ANCOM) between WT and miR-21KO mice after either sham operation or BDL for 3 days.

| **Feature** | **W** | **Taxon** | **Confidence** |
| --- | --- | --- | --- |
| 596f70447030c8a5e3ddf2bd3eadafa5 | 1055 | f__S24-7; g__; s__ | 0.9985 |
| 3bac14d2805bf995afe9d316a5777b1e | 1045 | f__Lactobacillaceae; g__Lactobacillus; s__helveticus | 0.7283 |
| 564828003ef8ce117a61d0098c6ff983 | 1042 | f__S24-7; g__; s__ | 0.9983 |
| 34ae37778971582a6242155115c276d5 | 1034 | f__S24-7; g__; s__ | 0.9975 |
| c1def48b42e159b33dd366fb124ea638 | 1024 | f__S24-7; g__; s__ | 0.9996 |
| f46712f8014a4facfe18e749aafd27fd | 975 | f__Neisseriaceae | 0.9999 |

**Table S2.** Primer sequences.

| **qPCR primers** | **primer Forward** | **primer Reverse** |
| --- | --- | --- |
| Zonula ocludens-1 (*Zo-1*) | 5′ GGA GCT ACG CTT GCC ACA CT 3′ | 5′ GGT CAA TCA GGA CAG AAA CAC AGT 3′ |
| Occludin-1 (*Ocldn-1*) | 5’ ATG TCC GGC CGA TGC TCT C 3’ | 5’ CTT TGG CTG CTC TTG GGT CTG TAT 3’ |
| Junctional adhesion molecule A (*Jam-a*) | 5’ TGT GGG ATT GGG CAA GAG TC 3’ | 5’ TAG AGT AGC TGG CAC CCC AT 3’ |
| Interleukin-1β (*Il-1β*) | 5′ TGC CAC CTT TTG ACA GTG ATG 3′ | 5′ TGA TGT GCT GCT GCG AGA TT 3′ |
| Toll like receptor-4 (*Tlr-4*) | 5’ TCC CTG CAT AGA GGT AGT TCC TA 3’ | 5’ CTT CAA GGG GTT GAA GCT CAG 3’ |
| Tumor growth factor-β (*Tgf-β*) | 5’ CTG CTG ACC CCC ACT GAT AC 3´ | 5’ GTG AGC GCT GAA TCG AAA GC 3’ |
| Tumor necrosis factor-α (*Tnf-α*) | 5’ AGG CAC TCC CCC AAA AGA TG 3’ | 5’ TGA GGG TCT GGG CCA TAG AA 3’ |
| Farnesoid X receptor (*Fxr*) | 5’ GGC TGC AAA GGT TTC TTC CG 3’ | 5’ CAG CCA ACA TCC CCA TCT CT 3’ |
| Leucine‑rich‑repeat‑containing G-protein-coupled receptor 5 (*Lgr5*) | 5’ CCT ACT CGA AGA CTT ACC  AGT 3´ | 5’ GCA TTG GGG TGA ATG ATA GCA 3’ |
| Olfactomedin-4 (*Olfm4*) | 5’ CAG CCA CTT TCC AAT TTC ACT G 3´ | 5’ GCT GGA CAT ACT CCT TCA CCT TA 3’ |
| Farnesoid X receptor (*Fxr*) | 5’ GGT GAA AAG GAC CTC TCG AAG TG 3’ | 5’ ATA GTC AAG GGC ATA TCC AAC AAC A 3’ |
| Macrophage inflammatory protein 2 (*Mip-2*) | 5′ GCT ACG AAC TGC CTG ACG G 3′ | 5′ GCT GTT ATA GGT GGT TTC GTG GA 3′ |
| D-lactate dehydrogenase (*D-ldh*) | 5' GAG GCT CTG AAG GCA GTT GT 3' | 5' GGA GGT TGA CAC CTG TGC AT 3' |
| Collagen-1α1 (*Col1α1*) | 5’ CTG ACT GGA AGA GCG GAG AG 3’ | 5’ GAC GGC TGA GTA GGG AAC AC 3’ |
| Cytochrome P450 7A1 (*Cyp7a1*) | 5’ CTG GGG GAT TGC TGT GGT AG 3’ | 5’ GCA CAG CCC AGG TAT GGA AT 3’ |
| *Lactobacillus reuteri* | 5’ GTA CGC ACT GGC CCA A 3’ | 5’ ACC GCA GGT CCA TCC CAG 3’ |

**Supplementary Figures**

**Figure S1.** Cohousing experiments of WT and miR-21KO mice. (A) Schematics of cohousing experiment. (B) *Lactobacillus sp.* present in cohoused mice experiments. Mean values were calculated as fold change versus each WT per house with error bars ± SEM. **p* < 0.05.

**Figure S2.** Observed ASVs for individual animals in both experiments. (A) WT and miR-21KO mice 3 days after either sham operation or BDL with minimum coverage 15238 reads. (B) Cohoused animals with the minimum coverage 21276 reads.
